# A Randomized Large-Scale Cross-Sectional Serological Survey of Hepatitis E Virus Infection in Belgian Pig Farms

**DOI:** 10.3390/microorganisms11010129

**Published:** 2023-01-04

**Authors:** Constance Wielick, Louisa Ludwig-Begall, Christel Faes, Stefaan Ribbens, Claude Saegerman, Etienne Thiry

**Affiliations:** 1 FARAH Research Centre, Veterinary Virology and Animal Viral Diseases, Department of Infectious and Parasitic Diseases, Faculty of Veterinary Medicine, University of Liège, 4000 Liège, Belgium; 2 Center for Statistics, Data Science Institute, Hasselt University, 3500 Hasselt, Belgium; 3 Animal Health Service Flanders (DGZ Vlaanderen), 8820 Torhout, Belgium; 4 Research Unit in Epidemiology and Risk Analysis Applied to Veterinary Sciences (UREAR ULiège), FARAH Research Centre, Department of Infectious and Parasitic Diseases, Faculty of Veterinary Medicine, University of Liège, 4000 Liège, Belgium

**Keywords:** hepatitis E, hepatitis E virus, pigs, swine, cross-sectional study, Belgium

## Abstract

Hepatitis E virus (HEV) is the causative agent of hepatitis E disease in humans. While sporadic HEV infections, which occur in industrialised countries and are typically due to HEV genotypes 3 or 4, are asymptomatic and self-limiting, a chronic form of the disease can lead to liver cirrhosis in immunocompromised individuals. Pigs share HEV 3 and 4 genotypes and are thus considered a major animal reservoir for human infection. A subset of animals has been shown to carry HEV particles at the age of slaughter, rendering raw or undercooked pig products potential vectors for human infection. To provide an overview of the current dissemination of HEV in Belgian pig herds, this study was designed as a randomized, robust, large-scale, cross-sectional, serological survey. HEV genotypes and subtypes recently circulating in Belgium (2020–2021) were investigated. Sample stratification as well as epidemiological investigation through the available demographic data of the sampled herds showed that HEV widely circulated in the Belgian pig population during this time and that a change in the circulating HEV strains may have occurred in the last decade. Herd size and type were identified as risk factors for HEV herd-seropositivity. Identifying farms at risk of being HEV-positive is an important step in controlling HEV spread and human infection.

## 1. Introduction

Hepatitis E virus (HEV), a small, non-enveloped, positive-sense, single-stranded RNA virus and member of the *Hepeviridae* family [1], is the causative agent of hepatitis E disease in humans. The World Health Organization (WHO) estimates that HEV causes around 20 million infections annually, of which, 3.3 million are symptomatic [2]. Hepatitis E virus genotypes 1, 2, 3, 4, and 7 are known to infect humans [1,3,4]. Hepatitis E virus 1 and 2 are endemic in developing countries and are responsible for large outbreaks of acute and fulminant hepatitis E disease epidemics (particularly severe in pregnant women) via contaminated drinking water (in Central and South Asia, and a large part of Africa) [5,6,7]. While sporadic HEV infections, which occur in industrialised countries (Europe and North America) and are typically due to HEV 3 or 4 [8], are frequently asymptomatic and self-limiting, a chronic form of the disease can lead to liver cirrhosis in immunocompromised individuals and are more commonly associated with neurological symptoms [7,9,10]. HEV seroprevalences in Belgium were recently estimated as 4.1% (95% CI 3.1–5.1) and 5.8% (CI 4.8–6.9) in 2006 and 2014, respectively [11]. Pigs share HEV 3 and 4 genotypes and are thus considered a major animal reservoir [12,13]. While transmission routes to and between humans remain to be fully elucidated, the oral route is increasingly implicated and mounting evidence shows a potential link between consumption of raw or undercooked pig and game meat and HEV infections [14]. As human infection is mostly asymptomatic and HEV 3 and 4 are found to be silently spread amongst pig populations [15,16,17,18,19], human HEV cases are likely largely underestimated. Hepatitis E virus is thus now considered an emerging public health concern, especially for immunocompromised individuals [9].

In the context of animal studies conducted around the world, pigs have been found to be highly HEV seroprevalent [15,16,17,18,19]. Importantly, a subset of animals has been shown to carry HEV particles at the age of slaughter, rendering raw or undercooked pig products potential vectors for human infection [20]. A first crucial step in the epidemio-surveillance of the virus is the identification of HEV-infected farms and possible risk factors for herd HEV-seropositivity. However, few published studies have included thorough cross-sectional analyses following well-defined and consensus-based guidelines. The epidemiology of European HEV infections thus remains insufficiently documented.

In Belgium, which is roughly separated into northern Flanders and southern Wallonia (each divided into five provinces), Animal Health Care Flanders (Dierengezondheidszorg Vlaanderen (DGZ)) and Animal Health Care Wallonia (Association Régionale de Santé et d’Identification Animales (ARSIA)), respectively, are designated by the Belgian Federal Agency for the Safety of the Food Chain (Agence Fédérale pour la Sécurité de la Chaîne Alimentaire/Federaal Agentschap voor de Veiligheid van de Voedselketen (AFSCA/FAVV)) to take charge of animal identification and health. A centralised database, the Belgian Identification & Registration System (SANITEL), is utilised as a computerised management system for the identification, registration, and monitoring of animals. Most professional pig production (active herds with at least 100 pigs) is situated in Flanders (90.79% of herds). The types of active pig farms can be defined according to the different rearing types registered in SANITEL and are comparable to those of other European countries. In the context of HEV, three farm types are of particular interest as they rear pigs which are subsequently sent to slaughter; they comprise mixed farrow-to-finish herds, closed farrow-to-finish herds, and slaughter pig herds. Farrow-to-finish herds raise piglets from birth to slaughter, while slaughter pig herds buy piglets (sometimes from multiple farms) and raise them from farrowing to fattening up until slaughter [21]. Closed farrow-to-finish herds only raise piglets born on the farm, while mixed farrow-to-finish herds also buy piglets born on other farms. Together, the herds corresponding to these three categories contribute to approximately 94.65% of the Belgian pig population [22]. The remaining 5.35% of herds consist of piglet rearing herds, breeding stock herds, sow rearing herds, and quarantine herds. A recent development in pig production is the increasing establishment of herds operating a free-range system in which pigs are allowed outside during rearing [23]; such herds are mostly located in Wallonia. Differences between free-range and more traditional professional systems include variations in farming intensity, pig-to-pig contact, environment (e.g., faecal distribution and cleaning), as well as differing possibilities of contact with the Belgian wild boar population [24]. Since all of these might feasibly influence HEV herd dynamics, special attention is paid to these types of farms in this study.

To provide an overview of the current dissemination of HEV in Belgian pig herds, this study was designed as a randomized, robust, large-scale, cross-sectional, serological survey and followed the checklist proposed by the Strengthening the Reporting of Observational Studies in Epidemiology (STROBE) statement [25]. A comparison with previous investigations [26] revealed potential changes in HEV distribution over the last 10 years; HEV genotypes and subtypes recently circulating in Belgium (2020–2021) were investigated. Importantly, sample stratification as well as epidemiological investigation through the available demographic data of the sampled herds allowed an initial risk profiling of Belgian pig farms. We show that HEV widely circulates amongst the Belgian pig population and that herd size and herd type influence the HEV-serological status of a pig herd. HEV genotype 3 subtypes a and c in the sera of young pigs are similar to isolates found in other European countries close to Belgium (isolated from pigs, humans, and wild boars) and resemble isolates found in Belgian HEV-infected patients. The study thus robustly completes and updates available data on the epidemiology of HEV infections in Belgium.

## 2. Materials and Methods

### 2.1. Sampling Protocol and Study Design

**Figure 1:**
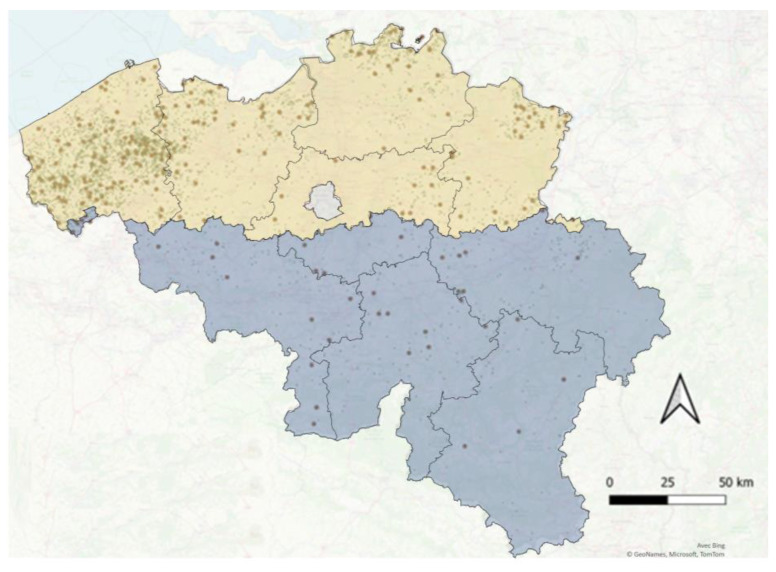
Geographical distribution of sampled pig farms in Belgium. All Belgian farms are represented by small green dots; sampled farms are represented by bigger brown dots; the Flemish region is shaded light yellow; the Walloon region is shaded blue.

**Figure A1:**
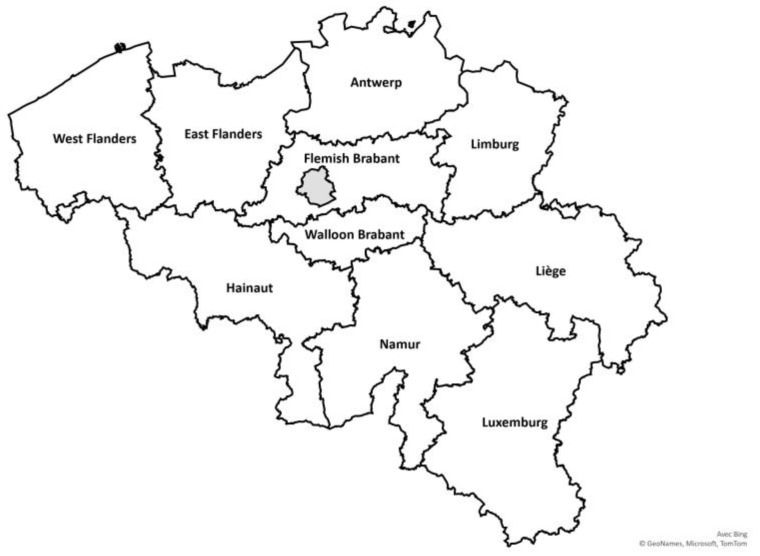
Map of Belgium showing the different provinces.

To ascertain an adequate sample size for the assessment of the HEV serological status in Belgian pig farms, a binomial law was applied with an expected herd-prevalence of 93% (with an absolute error of 5% and a confidence interval of 95% and using a finite population correction), this based on previous analyses performed by our team [26], and a sample size of 98 herds was determined. To allow for an optimised representation of the herds in each stratum, the sample size was then further extended and a total of 266 herds were selected. An up-to-date list of all pig herds active at the start of the study (11 May 2021) and encoded in SANITEL was anonymised and retrieved. The SANITEL database also provided descriptive demographic information for each of the herds (herd type, herd size, province, availability of a free-range system). Only herds housing at least 100 pigs (non-professional pig herds were thus omitted) and for which a minimum of twelve slaughter pig or six sow serum samples were in storage at the serum bank of the DGZ were retained. Herds were randomly selected using a stratified sample method (random generator Survey Toolbox [27]), according to their geographical localisation (in 10 Belgian provinces) and their herd type: mixed farrow-to-finish herd, closed farrow-to-finish herd, slaughter pig herd, and “other” (including quarantine farms, breeding stock farms). Each stratum, including the strata with few herds per stratum, was represented. Table 1 shows the distribution of the population and the sample herds in each stratum, while Figure 1 illustrates their geographical distribution in Belgium. Appendix A Figure A1 illustrates the geographical distribution of the Belgian provinces.

For each randomly selected herd, the testing strategy consisted of analysing six sera from adult sows. If no sera of sows were available (this was typically the case in slaughter pig herds), twelve sera from the oldest or heaviest pigs were selected. The selection of the serum samples was performed amongst those samples collected between 15 April 2020 and 30 April 2021 within the framework of the Belgian national Aujeszky’s disease control plan, in which, pigs are sampled annually or once every four months for free-ranging farms. The sampling method is defined by the ministerial decree of 23 July 2013 (Numac code: 2013018341 [28]). Per decree, this sampling must be randomly performed and must be evenly distributed throughout the herd, taking into account animals of different age groups. All samples are collected throughout a single day, unless otherwise specified (with a maximum 30-day interval). Sampled pigs should be at least ten weeks old (unless there are no older pigs present) and must have lived on the farm for at least one month. Approval from an ethics committee was not necessary, as no live animals were manipulated for the purpose of this study; only sera previously sampled for other purposes and subsequently stored in the serum bank of the DGZ were included.

Using the Animal Sample Size Calculator provided by the United States Department of Agriculture (USDA) Animal and Plant Health Inspection Service, the sample sizes of six or twelve sera were further validated; they were demonstrated to exceed the minimum sample size needed for disease detection, taking into account data on disease and test characteristics provided by Thiry et al., (2014): 70% or 30% seroprevalence, for six or twelve sampled sera, respectively, a test sensitivity (Se) of 98.55% [26], and a herd size of 4000 pigs as a worst-case scenario. According to the USDA’s Probability of Failure to Detect Diseased Animals Calculator, the probability to fail to detect a positive pig is less than 2% in the six-sera-scenario, and less than 3% in the twelve-sera-scenario (considering the same disease characteristics as described above) [29]. The sera were stored at −20 °C at the DGZ serum bank. Once transferred to the testing laboratory, they were stored at −80 °C until use.

### 2.2. Detection of Anti-HEV Antibodies

To reveal the presence of anti-HEV antibodies amongst sampled pigs, we used a double-antigen sandwich enzyme-linked immunosorbent assay (ELISA) (HEV ELISA kit 4.0V, MP Biomedicals). Detecting IgA, IgM, and IgG antibodies against HEV in serum from swine and other animal species, this assay has previously been shown to exhibit 98.55% Se and 40.91% specificity (Sp), using western blot as reference method [26]. Each serum was tested in duplicate. Cut off (CO) values were obtained following manufacturer’s instructions and were calculated for each assay; the mean optical density (OD) of each sample was calculated (S) and then divided by the assay CO to obtain the signal-to-cut-off value (S/CO). Results were considered positive if the S/CO was higher than 1. A herd was considered positive as soon as one sampled serum tested positive. Individual and herd prevalences were calculated with binomial confidence intervals of 95% (95% CI). A total of 2561 serum were tested for the presence of anti-HEV antibodies.

### 2.3. RNA Extraction

To reveal the presence of HEV RNA in young pigs, sera from pigs weighing less than 40 kg, and thus more likely to be viraemic than heavier/older animals [30], were chosen for RNA extraction and subsequent nested RT-PCR. RNA extractions were performed with the QIAamp Viral RNA mini kit (QIAGEN, Antwerpen, Belgium) and extracted RNA was treated using the TURBO DNA-free kit (Invitrogen, Merelbeke, Belgium) before storage at −80 °C until use. Each herd was processed separately and each serum was individually extracted. A negative extraction control was included for each herd (maximum twelve sera). One hundred and thirty µL of serum sample were used per assay. Ten µL of a murine norovirus (MNV) suspension (virus titre: 8 log10 TCID_50_/mL) were added as an internal extraction control. Extraction was considered successful if an 84 base pair (bp)—long MNV amplicon was revealed via RT-PCR (Table 2). RNA from a total of 392 sera was extracted.

### 2.4. Nested RT-PCR

Extracted RNA was tested using a nested RT-PCR (nRT-PCR) adapted from Huang et al. and able to detect HEV genotypes one to four [31,32]. Reverse-transcription and an external PCR amplifying a 731-bp long fragment within the HEV open reading frame 2 (ORF2) region, were performed as one step using the AccessQuick™ RT-PCR System (Promega, Leiden, The Netherlands) and a first set of external PCR primers (Table 2). Five µL of extracted RNA were used in a total reaction volume of 25 µL. The PCR parameters consisted of 45 min at 45 °C and 2 min at 94 °C, followed by 40 cycles of 1 min at 94 °C, 45 s at 47 °C, and 45 s at 68 °C, and a final elongation step (68 °C for 7 min). For the internal PCR amplifying a 348-bp long fragment, 2.5 µL of the external RT-PCR reaction product were used with the Taq DNA Polymerase kit with ThermoPol Buffer (New England BioLabs, Bioké, Leiden, The Netherlands) in a final volume of 25 µL and the second set of internal PCR primers (Table 2). Parameters of the second PCR were 2 min at 95 °C, followed by 40 cycles of 1 min at 95 °C, 45 s at 47 °C, and 45 s at 72 °C, and a 7-min-long final elongation at 72 °C.

For each RT-PCR or PCR performed, negative (water) and positive controls were included. The positive controls consisted of the WHO International Standard for HEV RNA Nucleic Acid Amplification Techniques-Based Assays (PEI code 6329/10) (Paul-Ehrlich Institut, Langen, Germany) as the external PCR positive control [33]) and a plasmid containing a HEV genotype 3 Kernow-C1 p6 clone [34,35] as the internal PCR positive control.

### 2.5. Sequencing and Phylogenetic Analysis of HEV Sequences

Positive nPCR-products of the correct molecular weight were excised from the gel and purified using the NucleoSpin^®^ Gel and PCR Clean-up kit (Macherey-Nagel, FilterService, Eupen, Belgium). The 348 bp-long amplicon was cloned using the pGEM^®^-T Easy Vector System (Promega, Leiden, The Netherlands). Recombinant plasmids were transformed into MAX Efficiency™ DH5α Competent Cells (Invitrogen, Merelbeke) and plated onto lysogeny broth agar plates containing ampicillin (100 µg/mL). After overnight growth at 37 °C, several clones were screened by PCR for the presence of the target fragment. Purification of plasmids was performed using the NucleoSpin^®^ Plasmid EasyPure kit (Macherey-Nagel, FilterService, Eupen, Belgium). One clone per plasmid was sent for sequencing to Eurofins Genomics (Ebersberg, Germany) using standard primers T7 and SP6. After trimming of primer sequences, 304 bp HEV sequences were analysed and aligned with a set of reference sequences proposed by Smith et al. [36] using MEGA version 6 [37], to determine genotypes and subtypes according to the classification of Lu et al. [38]. A set of additional sequences, comprising previously recovered sequences from human patients, swine, and wild boars in Belgium [24,26], and GenBank sequences corresponding to the best Basic Local Alignment Search Tool (BLAST) hits (https://blast.ncbi.nlm.nih.gov/Blast.cgi, accessed on 16 September 2022) for the sequences recovered in this study, was further included. A phylogenetic tree was constructed using the Maximum Likelihood method and Tamura-Nei model [39] and 1000 bootstrap replicates. Initial tree(s) for the heuristic search were obtained by applying the Neighbour-Joining method to a matrix of pairwise distances estimated using the Tamura-Nei model. A discrete Gamma distribution was used to model evolutionary rate differences among sites (five categories (+G, parameter = 0.1357)). This analysis involved 69 nucleotide sequences. All positions containing gaps and missing data were eliminated (complete deletion option). There was a total of 295 positions in the final dataset.

### 2.6. Statistical Analysis

The Pearson’s correlation coefficient and the non-parametric Spearman rank correlation coefficient were used to compare the randomly sampled herds with the complete Belgian pig population. The relationship between HEV-serological status (positive versus negative) and different exploratory variables (herd type, herd size, province, availability of a free-range system) was assessed using the odd ratios (OR) that were determined by logistic regression. When complete separation (zero cells) occurred, the Firth logit regression was used allowing inference of ORs and 95% confidence intervals (Heinze and Schemper, 2002). Statistical analyses were performed using STATA/SE Acad. 14.2 (Stata Corp., College Station, TX, USA). First, a univariate logistic regression analysis was performed. All variables showing a significant effect, with a *p*-value < 0.20 were selected (to allow for a more conservative model) and further analysed in a multivariate analysis. After conclusion of a backward stepwise approach, variables with a *p*-value < 0.05 were considered significantly related to herd HEV-serological status.

To estimate the within-herd and herd prevalence correcting for possible within-herd correlation, the number of animals tested in a herd and the ELISA assay’s Se and Sp, beta-binomial models were used as described by Faes et al. [40].

## 3. Results

### 3.1. HEV Widely Circulates amongst the Belgian Pig Population

**Figure 2:**
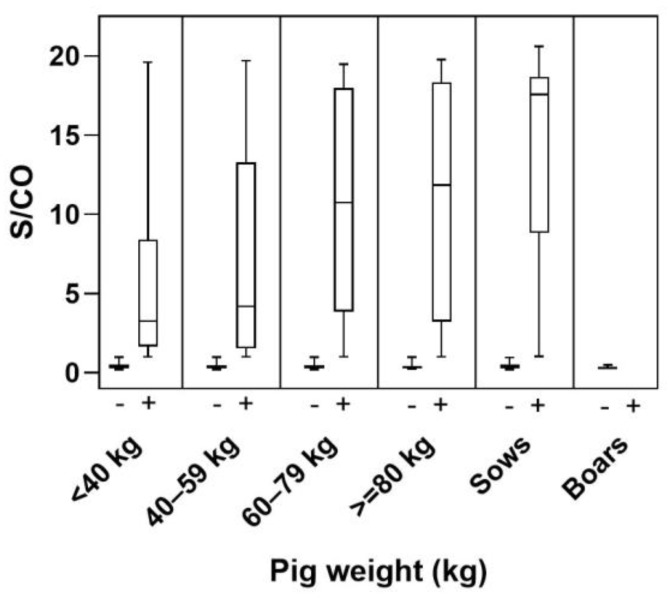
Distribution of the ELISA assay signal-to-cut-off values (S/CO) according to pig weight. S/CO values were obtained by dividing the mean optical density (OD) of each sample by the cut-off (CO) values of their respective assays. Results are positive if the S/CO is higher than 1. Quartiles and median S/CO values are represented.

**Figure 3:**
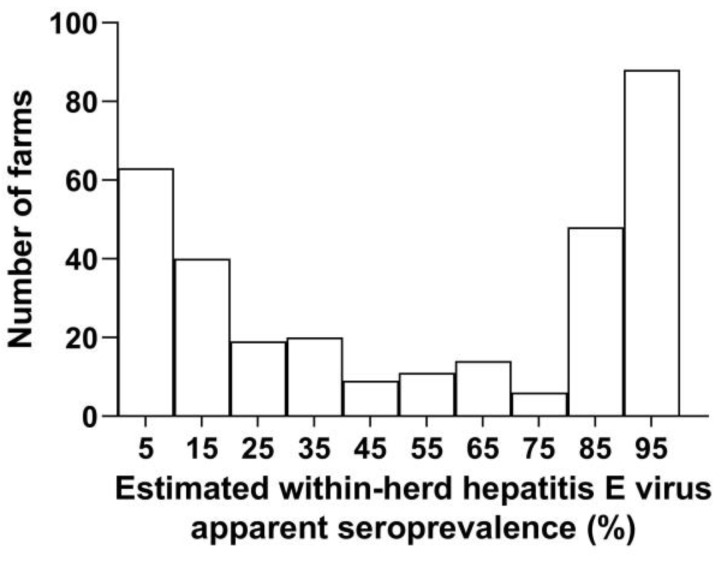
Distribution of the estimated within-herd hepatitis E virus apparent prevalence. Corrected estimates were obtained using a beta-binomial model, considering within-herd correlation and the size of the sampled herds. The ordinate represents the number of farms for different individual within-herd hepatitis E virus (HEV) prevalences (expressed in %; abscissa).

**Figure A2:**
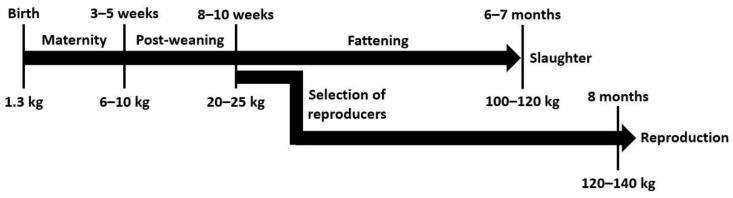
Timeline of a pig’s life in a typical Belgian pig production system. Probable weights and associated ages of animals at various pig production stages are indicated.

Using the Pearson’s correlation coefficient and the non-parametric Spearman rank correlation coefficient, the randomly selected herds were indirectly confirmed to constitute a representative sample of the Belgian pig population (*p*-value < 0.05). At the individual level, a total of 1213 (47.36% (95% CI = 45.43–49.30%)) pigs were shown to be positive for anti-HEV antibodies. HEV seroprevalence significantly differed with weight (*p*-value < 0.01) (Table 3). While seroprevalences seemed to increase with weight, pigs weighing less than 40 kg showed an individual seroprevalence close to that of heavier pigs (60 kg to ≥80 kg). The obtained S/CO values show that levels of absorbance increased with weight (Figure 2). Appendix A Figure A2 provides a guide to converting pig weights to an estimated age and the corresponding pig-production period. None of the tested reproductive boars was positive for anti-HEV antibodies. Apparent individual within-herd seroprevalence varied from 0% to 100%. Based on the beta-binomial model, it is observed that most of the herds have an estimated within-herd seroprevalence of either less than 10% or higher than 90% (Figure 3).

### 3.2. Herd Size and Type Influence the HEV-Serological Status of a Pig Herd

On a herd level, each of a total of 214 herds included at least one seropositive pig, demonstrating an overall herd seroprevalence of 80.45% (95% CI = 75.69–85.22%). Univariate analysis results showed HEV to circulate evenly in Belgium, revealing no significant differences in herd seroprevalence between the different Belgian provinces (*p*-value = 0.82) (Table 4). However, the herd HEV-serological status was shown to be affected by herd size and type (Table 5 and Table 6; *p*-values < 0.01). Seropositivity increased with the size of the herd (Table 5). HEV herd prevalence depended on herd type, specifically, slaughter-, closed and mixed farrow-to-finish pig herds, by ascending order of prevalence (Table 6). The presence of a free-range system did not seem to influence the herd serological status significantly (*p*-value = 0.12) (Table 7). Upon inclusion of the potential explanatory variables with a *p*-value < 0.20 from the univariate analysis (namely, herd type, herd size, and availability of a free-range system) in a multivariate analysis, increasing herd size and the two farrow-to-finish herd types were significant risk factors of herd HEV-seropositivity compared to “slaughter” and “other” herd types (*p*-values = 0.00808 and 0.00796, respectively) (Table 8, Appendix A Table A1). The multivariate analysis showed a 67% reduction in odds for mixed farrow-to-finish farms, a 90% reduction for other farms and an 86% reduction for slaughter farms, as compared to closed farrow-to-finish farms. For every ten-fold increase of herd size, the odds of being a HEV positive herd increased by a factor of 2.6.

Using a beta-binomial model, the results presented above were corrected with the test Sp and Se, the sampling designs (six versus twelve animals sampled), the herd sizes and the within-herd correlation. The estimates of true animal (within the herd) and true herd prevalences predicted are presented in Table 9. This table shows that the true prevalences might be lower than that we observe in the sample. Different sampling designs do not change much the estimated within-herd and herd prevalence. The correlation between animals within a herd is high (almost 60%; Table 9).

### 3.3. HEV Genotype 3 RNA Is Present in the Sera of Young Pigs in Belgium

**Figure 4:**
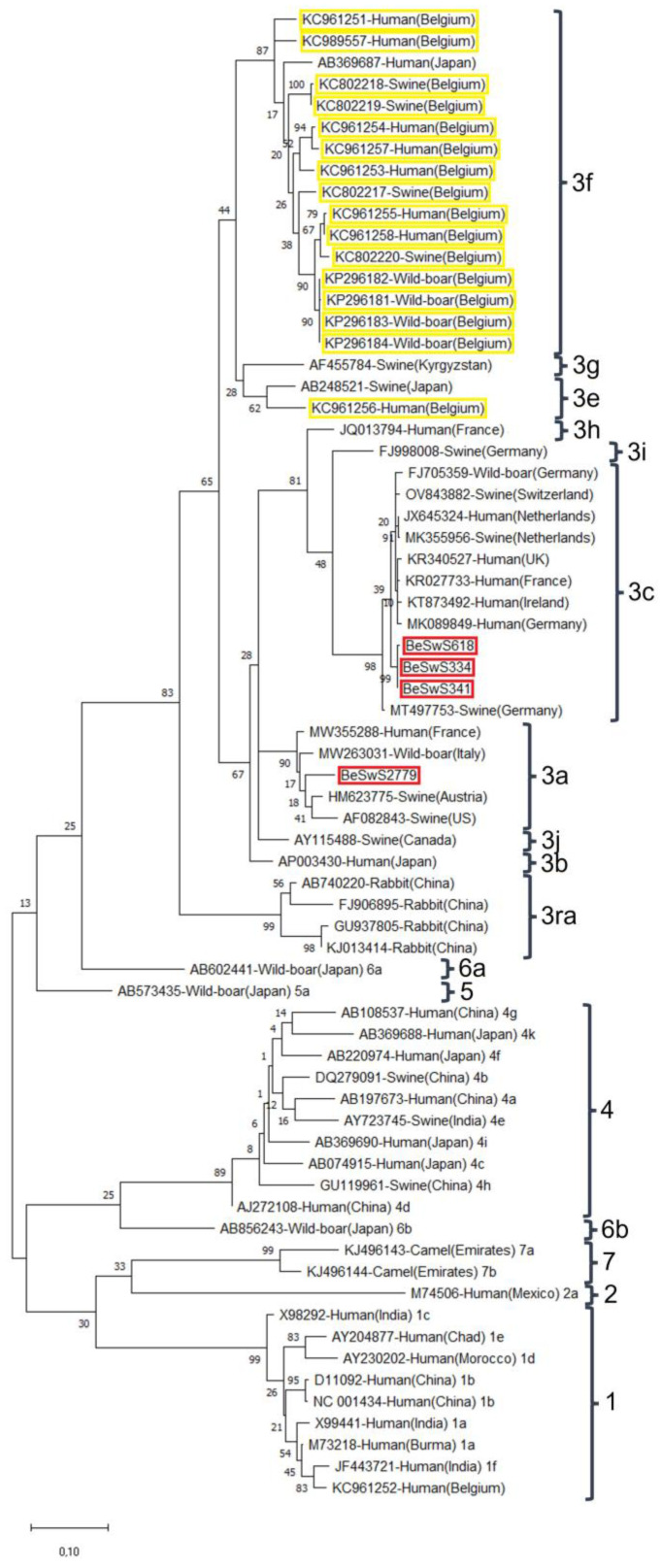
Maximum-likelihood phylogenetic tree including 64,304 bp-large Hepatitis E virus open reading frame 2 sequences. Four 304-base-pair-long hepatitis E virus (HEV) sequences recovered from swine sera in the present study (red boxes) were aligned with a set of reference sequences proposed by Smith et al. [36] using MEGA version 6 [37] to determine genotypes and subtypes according to the classification of Lu et al. [38]. A set of additional sequences was included, comprising previously recovered sequences from human patients, swine, and wild boars in Belgium [24,26] (yellow boxes) and GenBank sequences corresponding to previous best BLAST hits on the recovered sequences. Strains are noted as Accession number-Host (Country). Brackets designate grouped HEV strain association. The tree with the highest log-likelihood (−4930.57) is shown. The percentage of trees in which the associated taxa clustered together is shown next to the branches. The tree is drawn to scale, with branch lengths measured in the number of substitutions per site.

Following RNA extraction, nRT-PCR, and cloning, four HEV amplicons isolated from the serum of pigs weighing less than 40 kg were sequenced from both ends. All samples originated from seropositive slaughter pigs from the province of West Flanders. Two, BeSwS334 and BeSwS341, came from within the same pig herd. One isolate came from an HEV-seropositive pig, while the second came from an HEV-seronegative pig. These two sequences were highly similar to a third isolated from a seronegative pig (99% of nucleotide identity), BeSwW618, and clustered with HEV genotype 3 subtype c reference strains, which, in turn, have been shown to cluster with sequences isolated from pigs, wild boars, and humans from different European countries close to Belgium (Figure 4). The fourth sequence clustered with HEV 3 subtype a strains and was also isolated from a seronegative pig (Figure 4).

## 4. Discussion

Stratified sampling and serological analysis showed a widespread distribution of HEV infection in Belgian pig farms, with 47.36% and 80.45% apparent individual and herd seroprevalences, respectively. However, a beta-binomial model correcting for test Se and Sp (98.55% and 40.91%, respectively), the different sizes of the sampled herds, and the sampling design showed that these prevalences were likely overestimated and are indeed closer to 31.24% and 55.35%.

Hepatitis E seroprevalence and the detection of HEV genome in serum samples were investigated in Belgian pig farms over a decade ago using the same ELISA assay as the one implemented in the current study [26]. Between the two studies, individual prevalences among adult sows are similar: Thiry et al. found an individual seroprevalence of 73% (95% CI = 68.8–77.5%), while this study revealed 80.26% (95% CI = 77.12–83.40%) seropositivity [26]. Herd seroprevalences are less consistent, with 93% (95% CI = 89–100%) reported in 2010-2011 versus 80.45% (95% CI = 75.69–85.22%) ten years later. However, this study clearly highlights the influence of herd type on herd serological status. Since only herds housing adult sows were tested in 2010–2011, the study sample most probably comprised exclusively farrow-to-finish herds. Indeed, a direct comparison of 2022 farrow-to-finish herd seroprevalences with the overall seroprevalence reported by Thiry et al. yields similar results [26]. This suggests that herd HEV-seroprevalences remained almost unchanged over the last decade.

All reproductive boars tested seronegative in this study (Table 3). The rearing system for reproductive boars is different from the regular slaughter pig rearing system, in that boars are rapidly selected, undergo stringent biosecurity measures, and are sent to farms dedicated exclusively to their rearing for reproduction. Unfortunately, boars are not referenced in the database according to their weight. As very few boars were tested (18 boars from three “other” farms), no conclusions can be drawn here as to whether their joint seronegativity is attributable to their particular rearing system. Further investigation is thus needed in this branch of the porcine sector, especially as semen has been suggested as a potential route of transmission [41,42].

Individual seroprevalences tended to increase with the weight of pigs (Table 3). There was however an exception: pigs weighing less than 40 kg were shown to have a higher seroprevalence than pigs weighing 40–59 kg; at 44.9% this seroprevalence was similar to that of heavier slaughter pigs. This tendency has also been observed by several other authors worldwide, with comparable seroprevalences [43,44,45]. Analysis of S/CO values revealed an overall weaker absorbance signal of the ELISA assay in these younger pigs compared to older pigs (Figure 2). A possible explanation that consolidates these two observations is the presence of maternal antibodies transferred from sows to piglets during suckling. This hypothesis is supported both by reports of maternal antibody transfer (e.g., anti-HEV antibodies in piglets born from sows infected during gestation persisted for up to 2 months of age (circa 20 kg) and then faded after weaning [46]) and age-dependent fluctuation of sero-prevalences in a HEV-positive herd [30]. Our results thus show that sows are mostly seropositive and that piglets are protected from HEV infection for a few months, as suggested by Andraud et al. [47], delaying the age of first infection by HEV; this in turn can result in the presence of viraemic pigs at the slaughterhouse.

The size of pig herds, i.e., the number of housed pigs, typically varies over time (e.g., with departure of pigs to the slaughterhouse or the arrival of new piglets). The herd size registered in SANITEL thus represents the amount of available stabling space for pigs rather than the absolute amount of animals at any given time. As there is an order to the different size categories, the midpoints of their intervals are a good proxy to represent size differences between groups. Analysis of herd size as a continuous variable showed a correlation between herd seroprevalence and size; herd-seroprevalence increased by a factor 2.6 for every ten-fold increase of herd size (Table 8). Since higher animal concentrations typically facilitate the circulation of oro-faecally transmitted pathogens (such as HEV) in herds through concentration of viral particles in the environment [48], this result was not unexpected. Furthermore, in the case of HEV, herd size has been found to have an effect on HEV prevalence [49,50,51,52,53].

Types of pig farms were defined according to different rearing types. While these definitions may represent a somewhat oversimplified picture of the external biosecurity on individual farms, our results nevertheless showed a clear divergence of farrow-to-finish pig herds, with an increased herd-prevalence, as compared to all other types of herds (Table 6, Table 7 and Table 8). Biosecurity has been suggested to be a key factor for HEV introduction and circulation within a group of animals [49,54]. To pinpoint potential risk factors related to specific biosecurity measures, further investigations are needed.

Operating systems of free-range pig farms have been suggested to influence HEV herd dynamics [54,55]. Differences to more traditional professional systems include intensity of farming and pig-to-pig contact (head count per square metre), environmental differences (e.g., faecal distribution and cleaning) and possibilities of contact to the wild boar population [24]. Our own investigation into whether free-range systems pose an increased risk for HEV herd seropositivity showed that the presence of such a system did not significantly influence the HEV herd serological status (*p*-value = 0.12; Table 7 and Table 8). Since few herds matching the inclusion criteria of the sampling protocol implemented a free-range system, they were insufficiently represented to draw firm conclusions.

Amongst pigs weighing less than 40 kg, four 348 bp-long HEV sequences were isolated. This relatively low detection rate is probably due to the transient and short nature of HEV viraemia [56]. Furthermore, pigs in this category are typically at the pivotal period between the disappearance of maternal antibodies and the moment when they can be infected by HEV (weaning and mixing of piglets from different litters). Depending on individual herd management, such animals may be weaned at different ages. If animals are weaned later and/or maternal antibodies persist for a long time, young pigs may not yet have reached the viraemic phase of HEV infection at the time of sampling; this might explain the low amount of isolates found in this study. In addition, in the context of this retrospective study, we had no control over handling and storage of sera before their arrival at the laboratory. While this is unlikely to have had major implications for the serologic analysis, it may have compromised detection of viral particles in the sera and might explain the low serum RNA-positivity observed in this study. Finally, the Se of the nRT-PCR assay may be quite low, as this assay detects 31.6 50% pig infectious doses (PID_50_) [31] (corresponding to approximately 10^6^ HEV genomes/PID_50_ [57]). As the four isolated sequences belonged to HEV genotype 3 subtypes a and c, they did not cluster with sequences previously isolated in Belgium [24,26], of which all the sequences isolated from swine, humans, and wild boars belonged to subtype HEV 3f (Figure 4). These differences may be due to a combination of the low detection levels in both studies and might thus be attributed to coincidence. All sequences clustered with HEV isolated in other European countries close to Belgium and similar isolates found in Belgian HEV-infected patients [58]. However, a change in the HEV population cannot be completely ruled out. An increase of human infections due to HEV genotype 3 subtype c has, in fact, been observed in different European countries, including Belgium, over the last ten years [58,59]. Sampling faeces of these pigs may have increased the probability of finding positive samples, as the HEV excretion period is twice as long as viremia and starts earlier after infection [56]. The levels of viral RNA in faeces and livers are typically higher and thus more easily detected [60], and a larger proportion of pigs exhibit detectable viral RNA in their faeces [30] compared to their serum. However, this study was designed using an available large serum bank to allow for a randomised and representative sample of the Belgian pig population. Further investigations using a different study design might, however, provide further valuable information on the HEV strains circulating in Belgian pigs.

Potential biases were identified and addressed. The herd selection using a stratified randomisation and a computer random number generator is considered a low source of bias introduction. The representativeness of the herd sample was evaluated using both the Pearson’s correlation coefficient and the non-parametric Spearman rank correlation coefficient. Statistically, it was proved to be acceptable. Herds were selected amongst those for which at least twelve slaughter pig or six sow serum samples were available in the DGZ serum bank for the Belgian Aujeszky’s disease control plan. This serum bank contains sera from a large proportion (76.45% (3396/4442)) of the Belgian pig herd population housing at least 100 pigs. Of note, the herds targeted in this study were those housing at least 100 pigs, while nonprofessional herds were not accounted for. A small part of the herd population was thus voluntarily omitted.

The sera banked at the DGZ were sampled in the scope of the Aujeszky’s disease control plan. Sampling was thus performed, under legal obligation, by the herd’s official veterinarian and was carried out within a legal framework following a well-defined scheme (described in the Materials and Methods). This protocol ensures the sampling of pigs representative of herd serological status, in particular with regard to the fact that, if possible, the pigs should have been on the farm for at least one month. As sampling was performed by different field veterinarians, and not by one single investigator, slight variations in the classification of pig sample into the different categories might occur but cannot be considered as modifying the results of the study. Indeed, the results regarding seroprevalences and S/CO values according to the pig weight are in accordance with those reported by others [46].

A beta-binomial model was applied to correct for biases introduced by the sampling design and the ELISA assay (ELISA Se and Sp of 98.55% and 40.91%, respectively). As younger pigs were expected to have a lower seroprevalence, the six versus twelve sera approach depending on herd composition was chosen to increase the probability of finding a positive pig if no sera from adult sows were available. At the same time, the probability of detecting a positive pig was thus higher for the twelve-tested-sera herds, compared to the six-tested-sera herds. Table 9, however, shows that the true individual within-herd and herd prevalence may be considered similar, whether six or twelve sera were sampled. Furthermore, animals in a same herd may be considered as a subpopulation with the same environment, and thus, should have the same probabilities of being seropositive. An estimated high 60% within-herd correlation indicates that most of the variation between herds originates from within the herds. In other words, the higher the correlation, the higher the number of animals found positive if one animal is found seropositive. In further support of the six-tested-sera protocol, this also means that fewer sampled sera are needed to extrapolate herd HEV-status. Finally, the beta-binomial model shows an overestimation of the apparent herd-prevalence after correction. To our knowledge, this is the first time that such a model has been used in the context of HEV in pig farms and very few studies correct for the sampling design and the test Se and Sp. It is likely that published apparent individual and herd HEV-prevalences are generally overestimated, suggesting that HEV-free farms might be possible. An important implication is, that sufficient HEV-free farms may thus serve as a source of HEV-free pigs for a so-called HEV-free network, as proposed by a Scientific Opinion of the EFSA Panel on Biological Hazards (BIOHAZ) concerning the public health risks associated with HEV as a food-borne pathogen [61].

## 5. Conclusions

In conclusion, this work shows that HEV widely circulates in the Belgian pig population and herd HEV-seroprevalences remained almost unchanged over the last decade. Though herd seroprevalences remain high, they are likely overestimated. A change in HEV strains circulating among the Belgian pig population may have occurred, this concurrently with a switch observed in Belgian HEV-infected human patients. The conclusions drawn by this study in Belgium may be representative of other European countries, as the pig production system and swine husbandry in Western Europe are similar. Identifying farms at risk of being HEV-positive is an important step in controlling HEV spread and human infection. Therefore, these results set a baseline for future studies aiming to unravel the dynamic of HEV infection in pigs and for the potential development of mitigating measures for human infection.

## Figures and Tables

**Table 1:** Description of the herd stratification of the selected sample according to the Belgian pig herd population.

		Number of Herds by Herd Type (%)									
		Slaughter		Closed		Mixed		Other		Total	
Region	Province	Population	Sample	Population	Sample	Population	Sample	Population	Sample	Population	Sample
Flanders	West Flanders	1348 (29.97)	71 (26.69)	224 (4.98)	18 (6.77)	674 (14.98)	29 (10.90)	145 (3.22)	12 (4.51)	2391 (53.16)	130 (48.87)
	East Flanders	383 (8.51)	19 (7.14)	89 (1.98)	8 (3.01)	193 (4.29)	10 (3.76)	39 (0.87)	3 (1.13)	704 (15.65)	40 (15.04)
	Antwerp	282 (6.27)	13 (4.89)	75 (1.67)	5 (1.88)	149 (3.31)	7 (2.63)	29 (0.64)	3 (1.13)	535 (11.89)	28 (10.53)
	Limburg	163 (3.62)	8 (3.01)	48 (1.07)	5 (1.88)	102 (2.27)	8 (3.01)	23 (0.51)	0 (0.00)	336 (7.47)	21 (7.89)
	Flemish Brabant	56 (1.24)	5 (1.88)	24 (0.53)	2 (0.75)	35 (0.78)	3 (1.13)	3 (0.07)	1 (0.38)	118 (2.62)	11 (4.14)
Wallonia	Hainaut	89 (1.98)	8 (3.01)	21 (0.47)	2 (0.75)	18 (0.40)	1 (0.38)	4 (0.09)	3 (1.13)	132 (2.93)	14 (5.26)
	Liège	84 (1.87)	1 (0.38)	8 (0.18)	2 (0.75)	23 (0.51)	2 (0.75)	4 (0.09)	2 (0.75)	119 (2.65)	7 (2.63)
	Namur	60 (1.33)	3 (1.13)	10 (0.22)	2 (0.75)	10 (0.22)	2 (0.75)	3 (0.07)	0 (0.00)	83 (1.85)	7 (2.63)
	Walloon Brabant	14 (0.31)	2 (0.75)	4 (0.09)	2 (0.75)	8 (0.18)	1 (0.38)	0 (0.00)	0 (0.00)	26 (0.58)	5 (1.88)
	Luxembourg	22 (0.49)	1 (0.38)	7 (0.16)	1 (0.38)	25 (0.56)	1 (0.38)	0 (0.00)	0 (0.00)	54 (1.20)	3 (1.13)
Total		2501 (56.60)	131 (49.25)	510 (11.34)	47 (17.67)	1237 (27.50)	64 (24.06)	250 (5.56)	24 (9.02)	4498 (100.00)	266 (100.00)

Description of the herd stratification of the selected sample according to the Belgian pig herd population. Only herds housing at least 100 pigs are taken into account. Herd types comprise: slaughter pig herds (slaughter), closed farrow-to-finish herds (closed), mixed farrow-to-finish herds (mixed), and other herds, including quarantine farms and breeding stock farms (other). Absolute herd numbers as well as their relative percentages (in parentheses) are reported.

**Table 2:** Primers used in the nested RT-PCR to detect hepatitis E virus and a murine norovirus extraction control.

Primer Sequence (5′ → 3′)	Amplicon Length & HEV/MNV RNA Position	Final Concentration (nM)
**Hepatitis E virus**		
AATTATGCYCAGTAYCGRGTTG (F, external RT-PCR)	731 (5899–6629)	800
CCCTTRTCYTGCTGMGCATTCTC (R, external RT-PCR)		800
GTWATGCTYTGCATWCATGGCT (F, internal PCR)	348 (6184–6531)	800
AGCCGACGAAATCAATTCTGTC (R, internal PCR)		800
**Murine norovirus CW1**		
CGCTATGGATGCMAAGGA (F)	84 (389–472)	200
CCGATGTAGACAGAGTAATGGTA (R)		200

Primers used in the nested RT-PCR. Amplicon length and position on the hepatitis E virus or murine norovirus genome are given as base pairs. Abbreviations: HEV (Hepatitis E virus); MNV (Murine norovirus); Forward primer (F); reverse primer (R); reverse transcription (RT); nanomol (nM); Open reading frame (ORF). HEV sequence and positions are based on the complete genome sequence of genotype 3 Kernow-C1 p6 HEV clone (GenBank accession number JQ679013). MNV sequence and positions are based on the ORF1 sequence of murine norovirus CW1 strain (GenBank accession number AY228235).

**Table 3:** Apparent individual HEV seroprevalence according to pig weight.

	Number of Pigs by Weight (Prevalence (%) (95% CI))						
	<40 kg	40–59 kg	60–79 kg	≥80 kg	Sows	Boars	*p*-Value
Seropositive	183 (44.85 (39.96–49.82))	222 (28.43 (25.28–31.73))	201 (44.36 (36.94–45.88))	111 (44.40 (38.14–50.79))	496 (80.26 (76.90–83.33))	0 (0.00 (0.00–15.33))	<0.0001
Total	408	781	486	250	618	18	

Apparent individual hepatitis E virus (HEV) seroprevalence according to pig weight. Absolute pig numbers as well as their relative percentages (in parentheses) and exact binomial confidence interval (95% CI) are reported.

**Table 4:** Apparent herd hepatitis E virus seroprevalences in Belgian provinces.

Region	Province	Number of Seropositive Herds (Prevalence (%) (95% CI))	Total Number of Herds
Flanders	West Flanders	109 (83.85 (77.69–90.00))	130
	East Flanders	31 (77.50 (64.93–90.07))	40
	Antwerp	23 (82.14 (68.33–95.95))	28
	Limburg	16 (76.19 (58.55–93.83))	21
	Flemish Brabant	9 (81.82 (60.11–100.00))	11
Wallonia	Hainaut	9 (64.29 (40.55–88.02))	14
	Liège	6 (85.71 (60.57–100.00))	7
	Namur	6 (85.71 (60.91–100.00)	7
	Walloon Brabant	3 (60.00 (21.41–98.59))	5
	Luxembourg	2 (66.67 (14.83–100.00))	3
Total		214 (80.45 (75.83–85.07))	266

Apparent herd hepatitis E virus (HEV) seroprevalence depending on the Belgian province. Absolute herd numbers as well as their relative percentages (in parentheses) and exact binomial confidence interval (95% CI) are reported.

**Table 5:** Apparent herd hepatitis E virus seroprevalence according to herd size.

	Number of Herds by Herd Size (Prevalence (%) (95% CI))					
	101–200	200–500	501–1000	1001–2000	>2000	*p*-Value
Seropositive	3 (60.00 (14.66–94.73))	36 (72.00 (57.51–83.77))	46 (77.97 (65.27–87.71))	65 (77.38 (66.95–85.80))	64 (94.12 (85.62–98.37))	<0.01
Total	5	50	59	84	68	

Apparent herd hepatitis E virus (HEV) seroprevalence according to herd size. Absolute herd numbers as well as their relative percentages (in parentheses) and exact binomial confidence interval (95% CI) are reported.

**Table 6:** Apparent herd hepatitis E virus seroprevalence according to herd type.

	Number of Herds by Herd Type (Prevalence (%) (95% CI))				
	Slaughter	Closed	Mixed	Other	*p*-Value
Seropositive	96 (73.28 (64.85–80.63))	45 (95.74 (84.46–99.48))	57 (89.06 (78.75–95.49))	16 (66.67 (44.68–84.37))	<0.01
Total	131	47	64	24	

Apparent herd hepatitis E virus (HEV) seroprevalence according to herd type. Absolute herd numbers as well as their relative percentages (in parentheses) and exact binomial confidence interval (95% CI) are reported.

**Table 7:** Apparent herd hepatitis E virus seroprevalence according to the availability of a free-range system.

	Number of Herds (Prevalence (%) (95% CI))		
	Free Range System	No Free Range System	*p*-Value
Seropositive	19 (95.00 (75.13–99.87))	195 (79.27 (73.66–84.16))	0.12
Total	20	246	

Apparent herd hepatitis E virus (HEV) seroprevalence according to the availability of a free-range system. Absolute herd numbers as well as their relative percentages (in parentheses) and exact binomial confidence interval (95% CI) are reported.

**Table 8:** Effect of different demographic variables on the herd hepatitis E virus-serological status of 266 Belgian farms.

Variable	Category	N	Univariate Model		Multivariate Model	
			OR (95% CI)	*p*-Value	OR (95% CI)	*p*-Value
Type	Closed f-to-f	47	-	-	-	-
	Mixed f-to-f	64	0.36 (0.07–1.83)	0.219	0.33 (0.06–1.69)	0.184 *
	Other	24	0.09 (0.02–0.46)	0.004 ***	0.10 (0.02–0.55)	0.008 ***
	Slaughter	131	0.12 (0.03–0.53)	0.005 ***	0.14 (0.03–0.60)	0.008 ***
Size	-	266	3.65 (1.59–8.36)	0.002 ***	2.64 (1.10–6.34)	0.029 **
Free-range system	No	246	-	-	Not retained	
	Yes	20	4.97 (0.65–38.00)	0.122 *	Not retained	

Effect of different demographic variables (extracted from SANITEL) on the hepatitis E virus serological status of 266 farms. A univariate logistic regression analysis was performed. All variables showing a significant effect with a *p*-value < 0.20 were selected and further analysed in a multivariate logistic regression analysis. After conclusion of a backward stepwise approach, variables with a *p*-value < 0.05 were considered significantly related to herd HEV-serological status. Abbreviations: f-to-f (farrow-to-finish). * *p* < 0.20, ** *p* < 0.05, *** *p* < 0.01.

**Table 9:** Correction for within-herd and herd hepatitis E virus prevalence, taking ELISA sensitivity and specificity, herd size, and sampling design into account.

	Within-Herd Animal Prevalence (%) (95% CI)		Herd Prevalence (%) (95% CI)		Within-Herd Correlation (%) (95% CI)
	Apparent	True	Apparent	True	
Six sera sampled per herd	53.07 (48.26–57.64)	31.24 (31.20–31.27)	74.92 (69.95–79.27)	51.94 (49.35–54.60)	59.85 (49.35–64.63)
Twelve sera sampled per herd	53.12 (48.46–57.73)	31.24 (31.20–31.27)	80.37 (75.83–84.71)	58.33 (55.09–61.88)	59.93 (54.92–64.55)

Correction for within-herd and herd hepatitis E virus prevalence taking ELISA characteristics, herd size, and sampling design into account. Corrected estimates were obtained using a beta-binomial model, considering assay sensitivity and specificity, within-herd correlation and the size of the sampled herds. The herd hepatitis E virus prevalence was evaluated for different sampling designs: herd prevalence defined as at least one animal positive in six sampled pigs and herd prevalence defined as at least one animal positive in twelve sampled pigs.

## Data Availability

Data sharing not applicable.

## Appendix A

**Table A1:** Effect of the province on the herd hepatitis E virus-serological status of 266 Belgian farms.

Region	Categories within Province Variable	N	Univariate Model	
			OR (95% CI)	*p*-Value
Flanders	West Flanders	130	1.10 (0.37–3.21)	0.87
	East Flanders	40	0.73 (0.21–2.46)	0.61
	Antwerp	28	-	-
	Limburg	21	0.82 (0.19–3.53)	0.79
	Flemish Brabant	11	0.87 (0.14–5.40)	0.88
Wallonia	Hainaut	14	0.35 (0.08–1.52)	0.16 *
	Liège	7	1.09 (0.10–11.45)	0.82
	Namur	7	1.3 (0.13–13.37)	0.82
	Walloon Brabant	5	0.33 (0.04–3.53)	0.28
	Luxemburg	3	0.43 (0.03–5.78)	0.53

Effect of the geographic localisation on the hepatitis E virus (HEV)-serological status of 266 farms. A univariate logistic regression analysis was performed. * *p* < 0.20.
